# Plasmonic Nanohole Arrays on Top of Porous Silicon Sensors: A Win–Win Situation

**DOI:** 10.1021/acsami.1c07034

**Published:** 2021-07-23

**Authors:** Ruth F. Balderas-Valadez, Claudia Pacholski

**Affiliations:** Institute of Chemistry, University of Potsdam, Karl-Liebknecht-Str. 24-25, 14476 Potsdam OT Golm, Germany

**Keywords:** optical sensors, porous silicon, surface plasmon resonance, plasmonic nanohole arrays, bottom-up fabrication

## Abstract

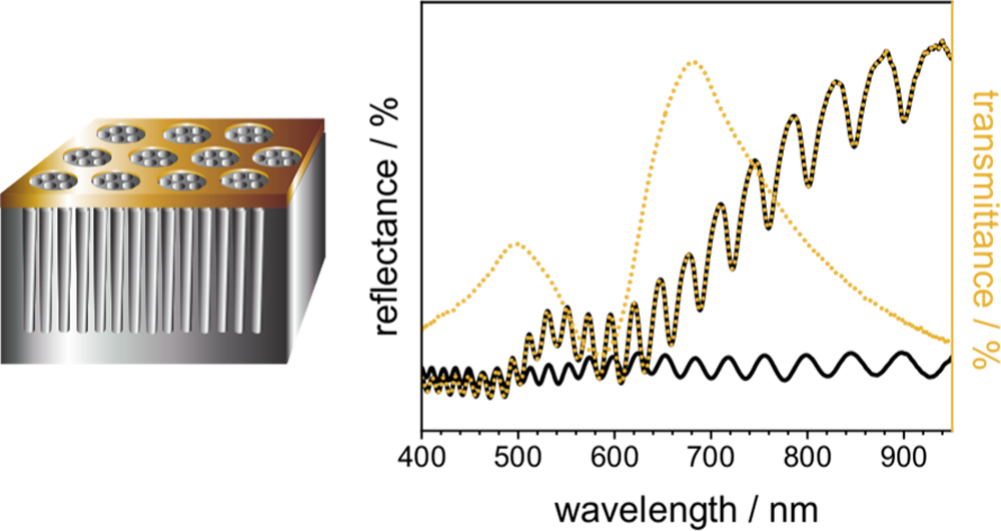

Label-free optical sensors are attractive candidates, for example, for detecting toxic substances and monitoring biomolecular interactions. Their performance can be pushed by the design of the sensor through clever material choices and integration of components. In this work, two porous materials, namely, porous silicon and plasmonic nanohole arrays, are combined in order to obtain increased sensitivity and dual-mode sensing capabilities. For this purpose, porous silicon monolayers are prepared by electrochemical etching and plasmonic nanohole arrays are obtained using a bottom-up strategy. Hybrid sensors of these two materials are realized by transferring the plasmonic nanohole array on top of the porous silicon. Reflectance spectra of the hybrid sensors are characterized by a fringe pattern resulting from the Fabry–Pérot interference at the porous silicon borders, which is overlaid with a broad dip based on surface plasmon resonance in the plasmonic nanohole array. In addition, the hybrid sensor shows a significant higher reflectance in comparison to the porous silicon monolayer. The sensitivities of the hybrid sensor to refractive index changes are separately determined for both components. A significant increase in sensitivity from 213 ± 12 to 386 ± 5 nm/RIU is determined for the transfer of the plasmonic nanohole array sensors from solid glass substrates to porous silicon monolayers. In contrast, the spectral position of the interference pattern of porous silicon monolayers in different media is not affected by the presence of the plasmonic nanohole array. However, the changes in fringe pattern reflectance of the hybrid sensor are increased 3.7-fold after being covered with plasmonic nanohole arrays and could be used for high-sensitivity sensing. Finally, the capability of the hybrid sensor for simultaneous and independent dual-mode sensing is demonstrated.

## Introduction

For decades, optical sensors have been studied to detect toxic and dangerous substances such as volatile organic compounds (VOC), explosives, pesticides, or antibiotics in food and also to monitor biomolecular interactions in order to develop safe and effective drugs.

Most prominent for the latter application are optical sensors based on surface plasmon resonance (SPR) in thin gold films, which were commercialized in the 1990s.^1^ Surface plasmons are collective oscillations of conduction band electrons and are highly sensitive to refractive index changes in the vicinity of the gold film. They can be excited in thin gold films by matching the momenta of the incoming light and the surface plasmon polaritons using prism coupling.^2^ Alternatively, a grating or a scattering structure can be integrated into the gold film for this purpose.^3^

Several advantages have been found in using gold films with integrated gratings such as periodic plasmonic nanohole arrays for sensor applications, ranging from lower production costs to easier optical readout in the transmission or reflection mode and enabling multiplexing.^4^ The sensitivity of these plasmonic nanohole arrays can be significantly improved, for example, with the geometrical layout of the plasmonic structure^5^ (figure of merit and spectral position of transmission/reflection bands) or using the supporting material.^6^ In the latter case, not only the refractive index of the supporting material dictates the optical signature of the sensor but the supporting material also defines the accessible surface area for capturing and detecting target analytes. Consequently, plasmonic nanohole arrays were not only fabricated on top of solid substrates but also combined with porous inorganic materials^7^ and polymers^8,9^ or even investigated as free-standing sensors.^10^ Using another optical sensor platform as a support for plasmonic nanohole arrays in order to provide a second sensing channel has not been published until now, to the best of our knowledge. However, first attempts to harness the advantages of combining plasmonic and interferometric sensors have recently been reported. For example, gold nanostructures showing localized SPR were combined with porous silicon (PSi) sensors.^11,12^

The appeal in using PSi for optical sensing is based on its high surface area, simple functionalization chemistry, and efficient fabrication by electrochemical etching, which give easy access to sophisticated optical structures such as photonic crystals.^13^ PSi has successfully been used as optical sensors for decades enabling, for example, the detection of DNA, antigen/antibody interactions,^14^ explosives, or VOCs.^15^ Different optical properties of PSi have been exploited for this purpose including specular reflectance and fluorescence. For improving the optical response of the PSi sensor, other materials were often deposited on top of the porous layer or filled into the pores.^16^ For example, gold nanoparticles were integrated into PSi sensors for interferometric sensing and for providing materials highly suitable for surface-enhanced spectroscopy.^17,18^

For realizing a hybrid sensor with two sensing channels, gold nanostructures were deposited onto the surface of PSi monolayers using a galvanic displacement reaction.^11^ Reflectance spectra of the resulting hybrid sensors were characterized by a fringe pattern resulting from the Fabry–Pérot interference at the borders of the PSi monolayer, in which a broad dip due to localized SPR (LSPR) in the nanostructured gold film could be noticed. Both the interference pattern and LSPR responded sensitively to refractive index changes in the surrounding medium. Similar results were presented for PSi rugate filters that were decorated on the surface and approximately in the upper third of the pores with wet-chemically synthesized gold nanoparticles.^12^ Nevertheless, all these hybrid sensors utilize gold nanoparticles showing LSPR, which limits the detection of analytes to a few nanometers (∼20 nm) above the gold surface. Plasmonic nanohole arrays probe the refractive index at 100–200 nm above the gold surface, facilitating highly sensitive detection of target analytes in a larger volume. Furthermore, almost all reported hybrid sensors were composed of PSi layers whose pores were at least partially filled with gold nanoparticles, preventing simultaneous dual-mode sensing of two target analytes. In addition, the pore size of the PSi was reduced by incorporation of gold nanoparticles, making the admission of biomolecules into the porous layer difficult. In a nutshell, these hybrid sensors leave a lot of room for improvement concerning the readout and analysis of their reflectance spectra, the design of the plasmonic structure, and the accessibility of the pores in the PSi layer—to name a few.

In this work, optical sensors based on plasmonic nanohole arrays, PSi monolayers, and the combination of both were fabricated and investigated in order to demonstrate the benefits of these hybrid sensors, which possess two transduction channels, provided by, namely, SPR and interferometry.

## Experimental Section

### Preparation of Plasmonic Nanohole Arrays

Plasmonic nanohole arrays were fabricated using a modified method from Quint and Pacholski,^19^ which is based on a combination of colloidal lithography and chemical gold deposition. In this work, polystyrene@poly-*N*-isopropylacrylamide (PS@poly-NIPAM) core–shell particles were utilized instead of pure poly-*N*-isopropylacrylamide microgels for preparing the colloidal mask. The PS@polyNIPAM core–shell particles were synthesized according to Kim and Ballauff.^20^ Details are given in the Supporting Information.

### Fabrication of PSi Sensors

PSi monolayers were prepared by wet electrochemical etching using a solution of ethanol and hydrofluoric acid (48%) in a volumetric ratio of 1:3. Silicon wafer pieces of ∼1.6 × 1.6 cm [boron doped (p-type), resistivity: <0.001 Ω cm, single-side polished, orientation: ⟨100⟩] were placed on top of an aluminum foil, which was part of a custom-made Teflon etching cell.^21^ After complete assembly of the Teflon etching cell, 4 mL of the electrolytic solution was poured into the cell, and a Pt ring electrode was immersed in the solution. Then, a current density of 442 mA cm^–2^ was applied for 30 s using a Kepco power supply in order to obtain the desired PSi monolayer. Freshly etched PSi samples were thoroughly washed with ethanol and immersed in pentane for 10 min. Then, the PSi samples were dried in a stream of N_2_.

To increase the stability of the fabricated PSi monolayers, they were thermally oxidized at 600 °C for 1 h using a muffle oven (Thermo Fisher Scientific). Furthermore, the surface of the oxidized PSi monolayers was functionalized with 3-aminopropyltriethoxysilane (APTES) by vapor deposition. For this purpose, PSi samples were placed in a desiccator together with a small container filled with 30 μL of APTES. A vacuum of 1 mbar was applied for 45 min. Finally, the APTES-functionalized PSi samples were thermally annealed at 110 °C for 1 h.

### Assembly of the Hybrid Sensor Composed of PSi and Plasmonic Nanohole Arrays

In order to detach the plasmonic nanohole array from the glass substrates, the samples were immersed in 0.1 M aqueous NaOH solution overnight. Then, the edges of the gold layer were scratched with a diamond tip of a glass cutter. The samples were removed from the basic solution and slowly immersed vertically in a MilliQ water bath (Figure S1a). This procedure lifted the plasmonic nanohole array layer from the glass substrate, and it floated on the surface of the water (Figure S1b).^22^ APTES-functionalized PSi samples were immersed in water (Figure S1c) and used to lift the plasmonic nanohole array layer from the air/water interface (Figure S1d). Finally, the dry sensors, consisting of the PSi structure and plasmonic nanohole array, were tempered for 24 h at 45° C in order to support the adhesion between the gold and the APTES-functionalized silicon surface.

### Characterization of Samples

#### Scanning Electron Microscopy

Scanning electron microscopy (SEM) was exploited for characterizing all samples regarding the size and the arrangement of nanoholes/pores and the thickness of the layers (gold/PSi). For this purpose, samples were equipped with a thin layer of carbon (∼5 nm) deposited using a combined system for carbon and sputter coating from Quorum Technologies (model Q150R ES). A scanning electron microscope from Zeiss (model Gemini ultra plus) was utilized for taking micrographs at an acceleration voltage of 3 keV with an InLens detector.

#### Optical Spectroscopy

A charged-coupled device (CCD) spectrometer (model Flame) from Ocean Optics, Inc. (USA) was utilized for collecting reflectance and transmittance spectra. For recording reflectance spectra, a bifurcated optical fiber was equipped with a microscope objective lens and connected to both the spectrometer and a tungsten light source. A spot of ∼1–2 mm^2^ was illuminated with the aid of the optical fiber. Reflectivity spectra were recorded every 15 s in a wavelength range of 400–1000 nm with a spectral acquisition time of 0.5 s and a total integration time of 20 ms, resulting from averaging 25 spectral scans. Reflectivity spectra were collected at normal incidence. In order to obtain reflectance spectra, the reflectivity spectrum of the respective sample was divided by a reflectivity reference spectrum, which was recorded previously from a clean silicon wafer. Transmittance spectra were collected by utilizing an appropriate optical setup obtained from Ocean Optics, Inc. (USA). The illuminated area on the samples was ∼5 mm in diameter. Transmittance spectra were recorded every 15 s in a wavelength range of 400–1000 nm with an integration time of 1 ms and 25 scans to average, resulting in an acquisition time of 25 ms.

### Flow Cell Experiments

A custom-made flow cell made of plexiglass was used for biomolecule adsorption/penetration experiments, whose layout has been reported previously.^23^ Briefly, the liquid was pumped through the flow cell via an inlet and outlet using a Perimax 12 SPETEC peristaltic pump and a flow rate of 0.57 mL/min. Reflectance/transmittance spectra were collected by guiding the light through the transparent flow cell using the already described optical setups.

### Data Analysis

Real-time monitoring of changes in the optical spectra of the sensors was achieved using IGOR Pro software (www.wavemetrics.com) equipped with the freely available online plugin Fringe_24_1 from M.J. Sailor (http://sailorgroup.ucsd.edu/software/index.html). A total of 20 optical spectra at each water/sucrose concentration were averaged to obtain the magnitude of EOT, Int_EOT_, and/or SPR corresponding to that concentration. The resulting values were determined for at least three individual sensors of each type (3 × PSi monolayer, 3 × plasmonic nanohole array, and 3 × hybrid sensor).

## Results and Discussion

### Characterization of the Hybrid Sensor

**Figure 1:**
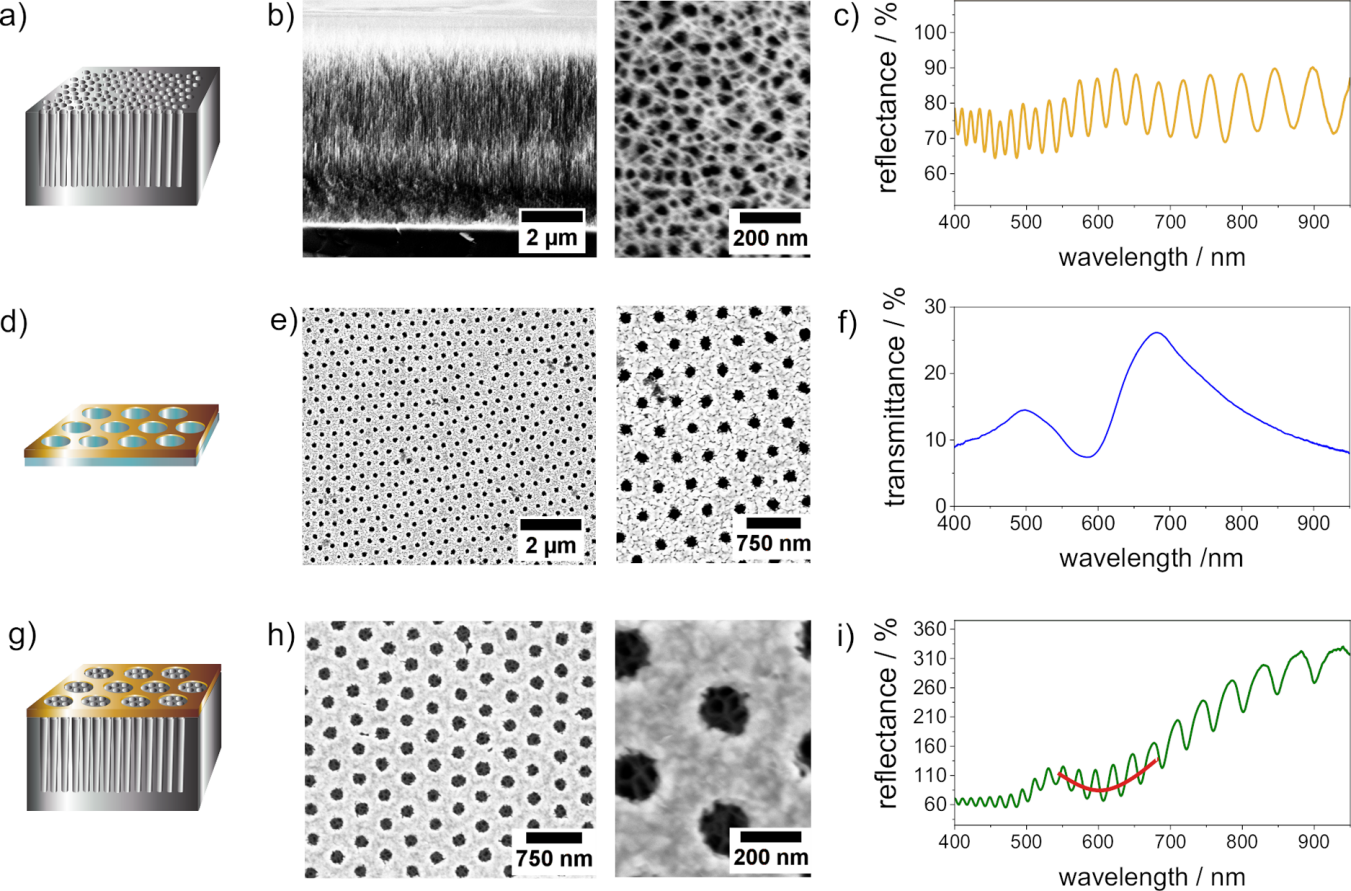
Fabricated optical sensors: (a) schematic of the PSi sensor, (b) scanning electron micrographs of PSi sensors, (c) reflectance spectrum of the PSi sensor, (d) schematic of the plasmonic nanohole array on the glass substrate, (e) scanning electron micrographs of the plasmonic nanohole array on the glass substrate, (f) transmittance spectrum of the plasmonic nanohole array on the glass substrate, (g) schematic of the hybrid sensor, (h) scanning electron micrographs of the hybrid sensor, and (i) reflectance spectrum of the hybrid sensor recorded in air.

In Figure 1, all optical sensors used in this study are displayed. PSi (PSi) sensors (Figure 1a) were prepared by wet electrochemical etching of highly doped, ⟨100⟩-oriented p-type silicon wafer pieces (<0.001 Ω cm) using a mixture of ethanol and hydrofluoric acid (48%) in a volumetric ratio of 1:3 and applying a current density of 442 mA cm^–2^ for 30 s. Freshly etched PSi sensors were thermally oxidized at 600 °C for 1 h to increase their stability in aqueous solutions.^24^

Figure 1b shows the scanning electron micrographs of PSi monolayers (cross-section and top view). The thickness of PSi monolayers was determined from cross-sectional scanning electron micrographs to be 6600 ± 100 nm. Top-view scanning electron micrographs revealed a sponge-like structure with non-uniform pore shapes and pore sizes of 22 ± 4 nm, which is typical of PSi fabricated from p-type silicon. A porosity of 84 ± 4% was obtained for the PSi monolayer using the spectroscopic liquid infiltration method.^21^ A representative reflectance spectrum of a PSi sensor collected in air is presented in Figure 1c, showing Fabry–Pérot fringes, which result from the interference of light rays reflected at the interfaces of the PSi monolayer.^25^ The spectral position of the interference maxima can be calculated using eq 1—the Fabry–Pérot relationship
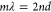
1where *m* = integer, λ = wavelength of incident light, *n* = average refractive index of the porous layer, and *d* = thickness of the porous layer.

Plasmonic nanohole arrays (Figure 1d) were fabricated using a modified bottom-up strategy developed by Quint and Pacholski.^19^ Briefly, polystyrene@poly-*N*-isopropylacrylamide core/shell colloids were self-assembled into highly ordered, loosely packed arrays and deposited on glass substrates. The array served as a mask for subsequent deposition of a gold film. Colloidal masks were removed by ultrasonication. Scanning electron micrographs of plasmonic nanohole arrays are displayed in Figure 1e. Established plugins in software ImageJ were utilized for determining key characteristics.^26^ Fabricated plasmonic hole arrays exhibited a high degree of order, which can be visualized by calculating the radial distribution function of overview scanning electron micrographs (Figure S2). The holes had a diameter of 173 nm ± 30 nm, and the lattice constant of the array was 399 ± 19 nm (*n* = 3, three SEM images of the nanohole array). The thickness of the gold film was determined to be 80 ± 7 nm by atomic force microscopy (Figure S3). In Figure 1f, a representative transmittance spectrum of a plasmonic nanohole array is displayed, which shows transmittance maxima at certain wavelengths resulting from excitation of SPR in the gold film by grating coupling. The spectral position of the transmittance maxima can be calculated using eq 2
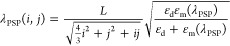
2where *L* is the lattice constant of the array, *i* and *j* are integers, and ε_d_ and ε_m_(λ_PSP_) represent the permittivity of the surrounding dielectric medium and the metal, respectively. The most pronounced transmittance maximum located at ∼680 nm can be assigned to the (1,0) gold/glass mode.^27^

Plasmonic nanohole arrays were lifted off the glass substrate by immersion in alkaline solution overnight and scratching the gold film.^22^ After slow immersion of the sample in water, the plasmonic nanohole array floated on the water surface, from where it was picked up with a PSi sensor functionalized with 3-aminopropyltrimethoxysilane (APTES). The resulting hybrid sensor composed of a plasmonic nanohole array on top of a PSi monolayer is presented in Figure 1g. Scanning electron micrographs reveal the successful transfer process without changing the properties of the single components (Figure 1h and S4). In Figure 1i, a reflectance spectrum of the hybrid sensor is displayed, in which both Fabry–Pérot fringes resulting from the PSi monolayer and a broad dip located at around ∼630 nm caused by SPR in the plasmonic nanohole array can be noticed, which is highlighted by a red line. The significant shift in the spectral position of the SPR of plasmonic nanohole arrays on top of a solid glass substrate (∼680 nm) and on top of PSi monolayers (∼630 nm) can be attributed to the change in the refractive index of the supporting material (ε_d_). Similar reflection spectra have already been reported for PSi structures with gold nanoparticles, in which the nanoparticles are either deposited on top of the PSi or partially infiltrated into the porous layer.^11,12,28^

### Performance of PSi Sensors with and without Nanohole Arrays

**Figure 2:**
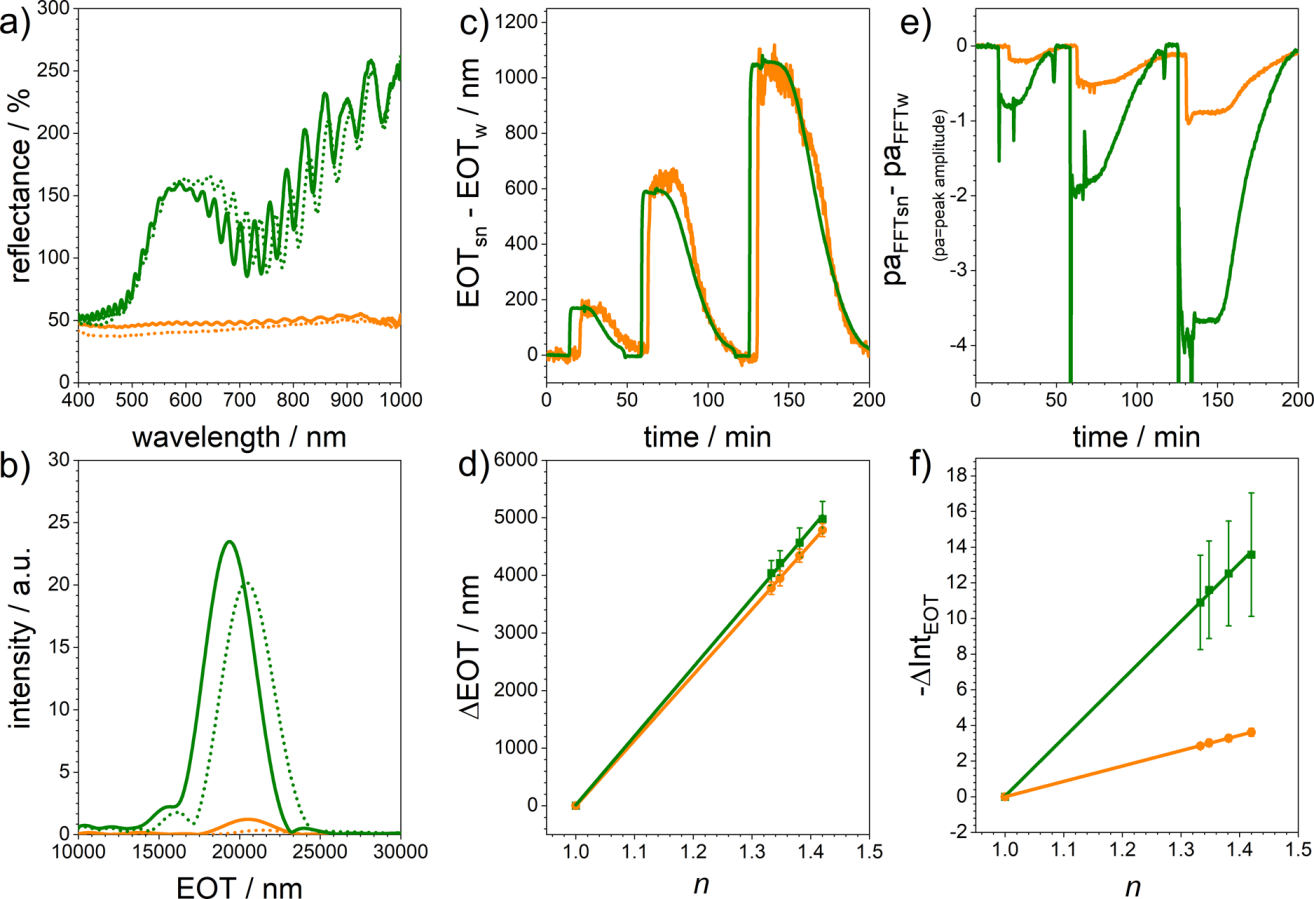
Sensor performance of PSi with (green lines) and without (orange lines) plasmonic nanohole arrays on top: (a) reflectance spectra recorded in MillQ water (solid line) and 50 wt % sucrose in MilliQ water (dashed line). (b) Fast Fourier transform of reflectance spectra, (c) changes in the EOT of the two sensors upon immersion in MilliQ water containing different amounts of sucrose (sequentially used solutions: MilliQ water, 10 weight % of sucrose in MilliQ water, MilliQ water, 30 weight % sucrose in MilliQ water, MilliQ water, 50 weight % sucrose in MilliQ water, and MilliQ water). (d) Changes in the amplitude of the FFT peaks of the two sensors upon immersion in MilliQ water containing different amounts of sucrose. (e) Sensitivity of the two sensors to refractive index changes calculated from EOT values (*n* = 3). (f) Sensitivity of the two sensors to refractive index changes calculated from FFT amplitude values (*n* = 3). Three individual sensors of each type were investigated.

Figure 2 shows the sensor performance of PSi sensors with (green lines) and without plasmonic nanohole arrays on top (orange lines). A direct comparison of their reflectance spectra is displayed in Figure 2a.

The significant differences between the reflectance spectra are immediately apparent. In addition to the presence of a dip in the interference pattern of the hybrid sensor, its reflectance spectrum is characterized by an enormous increase in reflectivity, which might be explained by the differences in the reflectivity of the top interface. Furthermore, the presented reflectance spectra were taken in MilliQ water (solid line) and 50 weight % sucrose in MilliQ water (dashed line). Both sensors, namely, PSi and hybrid sensors, respond to an increase in the refractive index (*n*_H_2_O_ = 1.3330 and *n*_50wt% sucrose_ = 1.4201) of the surrounding medium with a shift in the optical spectrum to higher wavelengths—as expected.

One of the most broadly applied methods for monitoring changes in the interference pattern of, for example, PSi sensors is based on applying a fast Fourier transform (FFT) to their spectra.^23,25^ By doing so, the effective optical thickness (EOT = 2 nL), which represents the refractive index of the porous layer multiplied with its thickness, can be directly obtained from the position of the FFT peak. The amplitude of the FFT peak gives information on changes in the refractive index contrast of the layer.^29^ In Figure 2b, the FFTs of the reflectance spectra shown in Figure 2a are displayed—using a wavelength range of 600–950 nm for analysis. The huge difference in the FFT amplitude is striking and can be attributed to the higher reflectivity of the plasmonic hole array made of gold in comparison to the PSi. A small shift in the EOT can be noticed between PSi sensors with and without plasmonic nanohole arrays on top. Both sensors respond to changes in the refractive index of the surrounding medium from H_2_O (*n* = 1.3330) to 50 weight % sucrose in MilliQ water (*n* = 1.4201) with a significant increase in EOT. This observation is in accordance with published results on PSi sensors decorated with gold nanoparticles.^12,17^

To determine the sensitivity of the interferometric sensors, flow cell experiments were carried out using MilliQ water for obtaining baselines and MilliQ water containing different amounts of sucrose (10% w/w of sucrose—*n* = 1.3478, 30% w/w of sucrose—*n* = 1.3812, and 50% w/w of sucrose—*n* = 1.4201) as analyte solutions. The optical response of both sensors to refractive index changes in the course of the experiment was monitored by determining the EOT from the FFT plots of the corresponding reflectance spectra (Figure 2c). Only small differences in the EOT values of the PSi with and without plasmonic nanohole arrays on top were observed. Most obvious was a decrease in the noise of the EOT values if a plasmonic nanohole array is present at the outer interface of the PSi layer. A partial coverage of the pores in the PSi by the gold layer does not seem to have any significant influence on the penetration of the liquid into the pores under the selected conditions. The sensitivity of the EOT shift of both sensors was determined by plotting the difference in the position of the EOT in the FFT in air and in solution (ΔEOT = EOT_*n*≠1_ – EOT_*n*=1_) versus the refractive index of the surrounding medium (Figure 2d). An expected linear relationship between the EOT position and refractive index can be noticed, that is, with increasing refractive index, the EOT position shifted to higher values. The sensitivities were calculated by applying a linear fit and determining its slope (=sensitivity), which yields values of 11,959 ± 150 and 11,378 ± 24 nm/RIU for PSi monolayers with and without plasmonic nanohole arrays on top, respectively. There is no significant difference in the sensitivity of the two sensors. Similar results have been reported for PSi sensors whose top surface was covered with gold nanoparticles.^11^

Figure 2e shows changes in the amplitude of the FFT peak when MilliQ water and various sucrose solutions flowed alternately over the sensors. A drastic increase in the response of the PSi sensor after covering the top with a plasmonic nanohole array can be seen, which might be related to the higher refractive index contrast between gold and the surrounding medium in comparison to that in PSi immersed in liquids. This observation was already reported by Dronov et al. when investigating porous alumina covered with a thin layer of platinum.^30^ To evaluate the sensitivity of the FFT peak amplitude to refractive index changes, the difference between its magnitude in air and in solution (ΔInt_EOT_ = *I*_EOT *n*≠1_ – *I*_EOT *n*=1_) was calculated. A plot of the FFT peak amplitude vs the refractive index of the medium is displayed in Figure 2f. Again, a linear relationship was obtained, and bulk refractive index sensitivities of 33 ± 1 and 9 ± 0 in intensity per RIU were determined for PSi with and without plasmonic nanohole arrays on top, respectively. Three separately manufactured sensors were examined for these experiments (*n* = 3). The standard deviation of these three measurements was calculated and is given as error bars in the graph. The raw data can be found in the Supporting Information (Figures S6 and S7). In addition, the reproducibility of the optical response of one sensor (PSi and hybrid sensors) for different sucrose solutions is shown in Figure S8. Here, the error bars represent the standard deviation for changes in the optical signal corresponding to one sucrose solution (shown in black, almost invisible due to their small values). However, these values depend on the wavelength range of the reflectance spectrum, which is chosen for calculating the FFT (here: 600–950 nm).

### Performance of Nanohole Arrays on Glass Substrates and PSi

**Figure 3:**
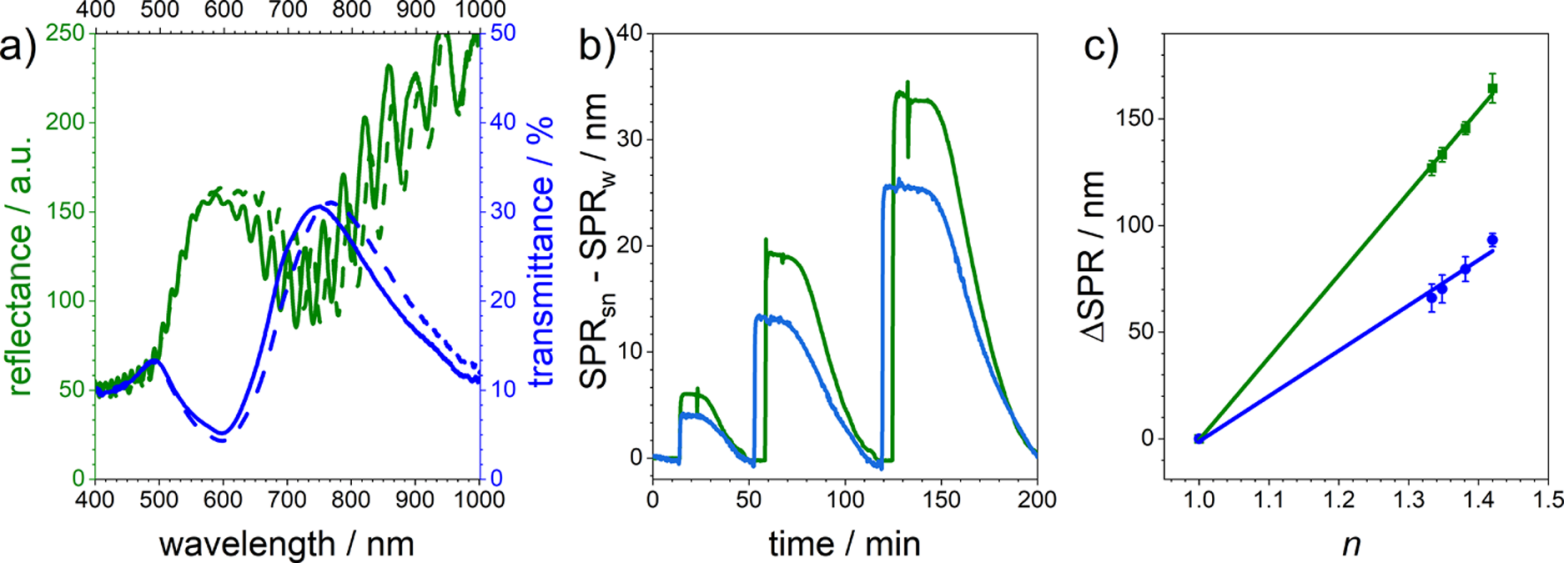
Sensor performance of plasmonic nanohole arrays with the solid glass substrate (blue lines) and PSi underneath (green lines): (a) transmittance and reflectance spectra recorded in MilliQ water (solid line) and 50 weight % sucrose in MilliQ water (dashed line), (b) changes in the spectral position of the SPR maximum/minimum of the two sensors upon immersion in MilliQ water containing different amounts of sucrose (sequentially used solutions: MilliQ water, 10 weight % of sucrose in MilliQ water, MilliQ water, 30 weight % sucrose in MilliQ water, MilliQ water, 50 weight % sucrose in MilliQ water, and MilliQ water), and (c) sensitivity of the two sensors to refractive index changes.

In Figure 3, the optical sensor performances of plasmonic nanohole arrays on glass substrates (blue lines) and PSi monolayers (green lines) are compared. Reflectance and transmittance spectra of plasmonic nanohole arrays on top of PSi monolayers and glass substrates are shown in Figure 3a. The coincidence of the transmittance maximum [(1,0) gold/glass mode] of the plasmonic nanohole array on glass with the dip in the reflectance spectrum of the PSi sensor covered with the same plasmonic structure is evident and indicates the same origin—SPR in the gold film. Moreover, spectra of both sensors recorded in MilliQ H_2_O (solid lines) and 50 wt % aqueous sucrose solution (dashed lines) are shown. In both cases, the spectral position of the plasmonic peak increases with a rise in the refractive index of the surrounding medium. In Figure 3b, a plot of the spectral position of the SPR peaks of both sensors versus time is displayed for a flow cell experiment in which the sensors were exposed to MilliQ water and sucrose solutions with various concentrations. A higher response of the plasmonic nanohole array on top of PSi to refractive index changes can be easily perceived.

Determination of the sensitivities of the two sensors was achieved by calculating the slopes of the linear relationships between the spectral positions of the SPR peaks in the reflectance and transmittance spectra (Figure 3c). Three separately fabricated sensors were examined for these experiments (*n* = 3). The standard deviation of these three measurements was calculated and is given as error bars in the graph. The raw data can be found in the Supporting Information (Figure S9). Plasmonic nanohole arrays on top of PSi monolayers had a sensitivity of 386 ± 5 nm/RIU, whereas sensors prepared on glass substrates had sensitivities of 213 ± 12 nm/RIU. The higher sensitivity of the hybrid sensors might be based on the larger accessible surface area for sensing (PSi vs solid glass).^10,31^

### Demonstration of Dual-Mode Biosensing

**Figure 4:**
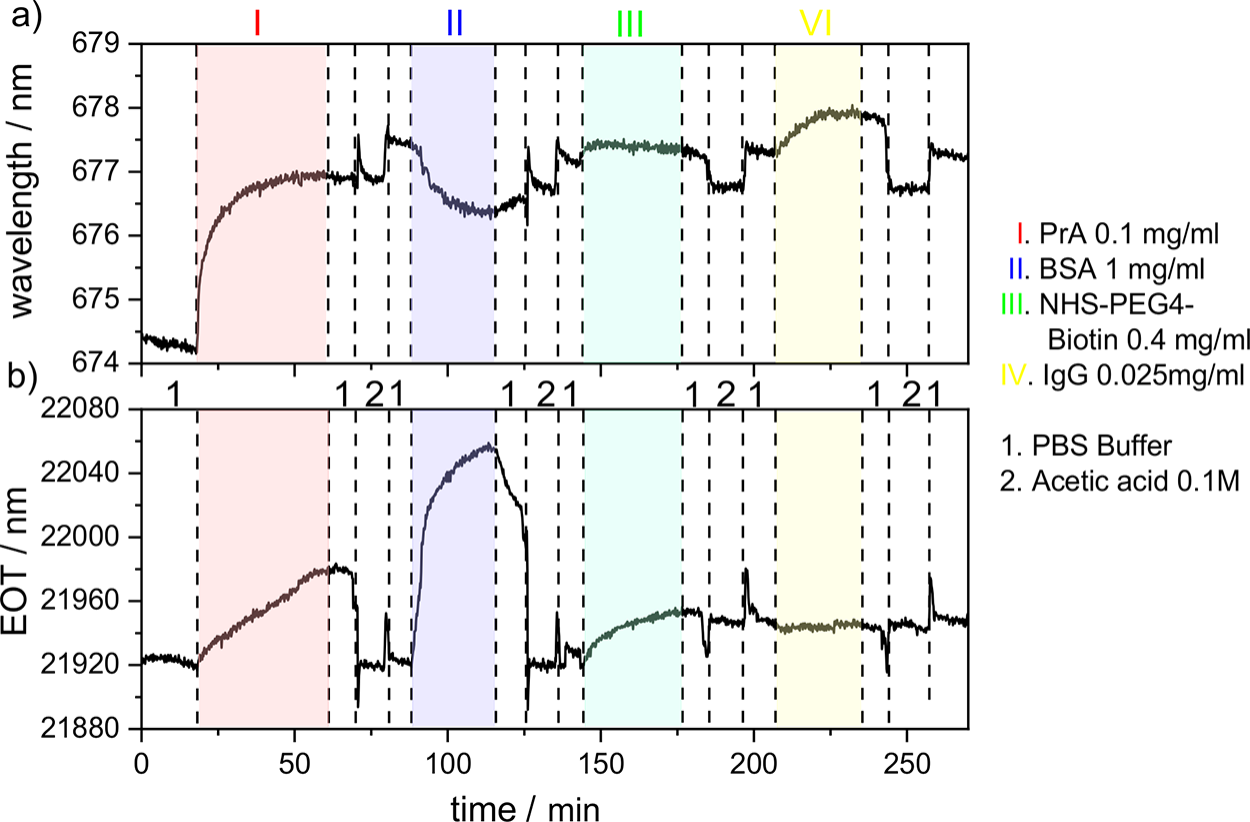
Dual-mode sensing with a hybrid sensor composed of a plasmonic nanohole array on top of PSi monolayers: (a) optical response of the plasmonic nanohole array represented by changes in the spectral position of the SPR and (b) optical response of the PSi monolayer represented by changes in the EOT. The following solutions were subsequently flowed over the hybrid senor surface: PBS pH = 7.4, 0.1 mg/mL protein A in PBS, PBS pH = 7.4, 0.1 M acetic acid, PBS pH = 7.4, 1 mg/mL BSA in PBS, PBS pH = 7.4, 0.1 M acetic acid, PBS pH = 7.4, 0.4 mg/mL NHS–PEG_4_–biotin in PBS, PBS pH = 7.4, 0.1 M acetic acid, PBS pH = 7.4, 0.025 mg/mL 0.025 mg/mL rabbit immunoglobulin G, PBS pH = 7.4, 0.1 M acetic acid, and PBS pH = 7.4, 0.025 mg/mL.

Finally, the capability of the hybrid sensor composed of a PSi monolayer covered with a plasmonic nanohole array for dual-mode sensing was investigated by performing flow cell experiments. For this purpose, the sensor surface was subsequently exposed to different solutions of biomolecules, namely, protein A, bovine serum albumin (BSA), functionalized biotin, and rabbit IgG. Figure 4 shows the optical responses of the two sensing channels of the hybrid sensor to changes in the surrounding medium, which have been simultaneously recorded from the same sensor spot. After establishing a stable baseline in PBS buffer (∼18 min), 0.1 mg/mL protein A in PBS is flowed over the sensor surface, leading to shifts in the spectral position of the SPR of the plasmonic nanohole array and in the EOT related to the PSi monolayer (from ∼18 to ∼61 min). Upon exposure of the sensor to PBS (from ∼61 to ∼72 min) and 0.1 M acetic acid solutions (from ∼72 to ∼81 min), the protein A was successfully removed from the PSi layer but remained adsorbed on the gold surface. Protein A has a strong affinity to surfaces made of gold and cannot be easily detached.^32,33^ In contrast, PSi functionalized with APTES has a positive charge in acidic media, and its electrostatic repulsive interaction with protein A, which is also positively charged (at this pH value), facilitates the removal of protein A from the PSi layer. Similar observations have already been described for BSA.^34^ Afterward, PBS buffer was flowed over the sensor in order to establish a new baseline (from ∼81 to ∼88 min). Subsequent to immersion of the hybrid sensor in PBS buffer, a solution of 1 mg/mL BSA in PBS induced only an optical response of the PSi layer (∼88 to ∼116 min). The plasmonic nanohole array already covered with protein A responded to the presence of BSA with a spectral shift in the SPR to shorter wavelengths, indicating a decrease in the refractive index. Thus, as expected, no binding between BSA and protein A was detected. The shift is most likely related to the inaccuracy of the fit of the relatively broad SPR signal used to determine the position of the SPR signal on the wavelength scale. In other experiments, this optical response could not be detected (Figure S10). After the removal of BSA from the PSi layer by flowing PBS (from ∼116 to ∼126 and from ∼136 to ∼144 min) and 0.1 M acetic acid (from ∼126 to ∼136 min) over the senor surface, the sensor was exposed to a solution of NHS–PEG_4_–biotin in PBS (∼144 to ∼175 min). This molecule can bind to primary amine groups via its NHS group (NHS: *N*-hydroxysuccinimide) and reacts with amine groups present in the PSi layer due to its functionalization with APTES and with the amine groups of protein A, which is adsorbed to the plasmonic nanohole array.^35^ In this reaction, NHS is released, and PEG_4_–biotin is covalently bound to the amine groups of protein A and APTES.^36^ Consequently, changes in the SPR spectral position and EOT were expected. Indeed, a small shift in the spectral position of the SPR signal is evident. However, due to the coverage of the plasmonic nanohole array with protein A, only a small change in the refractive index in this protein layer is achieved by the binding of the small-molecule PEG_4_–biotin to some of the accessible amine groups in protein A. In the PSi layer, the free amine groups of the self-assembled monolayer of APTES are all readily accessible to the binding of PEG_4_–biotin, resulting in a larger change in the refractive index and consequently a significant change in the EOT. Afterward, PBS buffer (from ∼175 to ∼184 and from ∼196 to ∼207 min) and 0.1 M acetic acid (from ∼184 to ∼196 min) were flowed over the sensor in order to establish a new baseline. Due to the PEG_4_–biotin covalently bound to the APTS-functionalized PSi surface, the EOT signal did not return to the baseline obtained before the sensor was exposed to NHS–PEG_4_–biotin. Finally, the hybrid sensor was immersed in a solution of 0.025 mg/mL rabbit IgG, which selectively binds to protein A (from ∼207 to ∼236 min). Indeed, protein A is often used for functionalizing biosensor surfaces because it allows for directed immobilization of immunoglobulins (IgGs).^37,38^ Only the plasmonic nanohole array covered with protein A showed an optical response, indicating that rabbit IgG did not infiltrate into the PSi layer and showed a typical association and dissociation to protein A adsorbed to the plasmonic nanohole array. The covalent binding of a small amount of PEG_4_–biotin obviously did not prevent this specific biomolecular interaction. Degradation of the PSi sensor or removal of APTES from its surface was not detected throughout the biosensing experiments. The interactions between biomolecules, buffer, APTES, and the PSi surface are rather complex, and the stability of surface functionalization using APTES has been studied in detail previously.^39^

In summary, three different optical sensors–plasmonic nanohole arrays on glass substrates, PSi monolayers, and their combination (plasmonic nanohole arrays on top of PSi monolayers)—were fabricated and characterized, and their sensing performance was investigated. Plasmonic nanohole arrays were obtained using a bottom-up strategy and showed extraordinary transmission of light provided by SPR in the gold layer. In their optical transmittance spectra, a strong maximum can be noticed, which is related to the (1,0) gold/glass mode and responded to refractive index changes with a sensitivity of 212.6 ± 11.6 nm/RIU. By transferring plasmonic nanohole arrays on top of PSi layers, the accessible surface area for sensing was enlarged, and the sensitivity of the plasmonic structure significantly increased to 386.3 ± 5.4 nm/RIU. The same trend was observed for detecting the adsorption of proteins to the sensor surface. Furthermore, the underlying PSi monolayer can be exploited for interferometric sensing by monitoring changes in its EOT, and its sensitivity was determined to be 11,958.6 ± 149.6 nm/RIU. In this case, the presence of the plasmonic nanohole array on top did not have an influence on the sensitivity of the interferometric sensor, which had a sensitivity of 11,377.7 nm/RIU ± 23.6 without the plasmonic structure on top. However, if no changes in the EOT were considered except variations in the amplitude of the EOT peak, a drastic gain was found in sensitivity from 8.6 ± 0.1 to 32.7 ± 0.5 per RIU for PSi sensors without and with plasmonic nanohole arrays on top, respectively. Hence, the increase in the sensitivity of PSi sensors covered with gold nanostructures might be traced back solely to an enhanced reflectivity of the top layer. Furthermore, dual-mode sensing of the hybrid sensor was successfully demonstrated by simultaneously monitoring the optical response of both channels, namely, SPR (plasmonic nanohole array) and EOT changes (PSi monolayer), upon exposure to different biomolecule solutions.

The benefits of combining plasmonic and interferometric sensor platforms can be manifold, ranging from increased sensitivities and the independent utilization of different optical transduction methods for monitoring refractive index changes to the vast opportunities for functionalizing their surfaces with different capture probes. These kinds of sensors pave the road to multiparametric sensors and can be easily miniaturized due to their bottom-up fabrication strategy.

## Supporting Information Available

Experimental details, radial distribution function, and atomic force microscopy images of plasmonic nanohole arrays on solid glass substrates, additional SEM images of plasmonic nanohole arrays on top of the PSi monolayer, detection of biomolecules using plasmonic nanohole arrays, and details of the biosensing experiment (PDF)

## Abbreviations

APTES: 3-aminopropyltriethoxysilane
EOT: effective optical thickness
FFT: fast Fourier transform
LSPR: localized surface plasmon resonance
polyNIPAM: poly-*N*-isopropylacrylamide
PSi: porous silicon
RIU: refractive index units
SEM: scanning electron microscopy
SPR: surface plasmon resonance
