# Supplementary material for: Plasmonic Nanohole Arrays on Top of Porous Silicon Sensors: A Win–Win Situation
Source: ACS Appl Mater Interfaces. 2021 Jul 23;13(30):36436–44. doi: 10.1021/acsami.1c07034 (PMC10015452; doi:10.1021/acsami.1c07034)
Supplement: Supplementary file 1 — am1c07034_si_001.pdf [file am1c07034_si_001.pdf]

Supporting Information

Plasmonic nanohole arrays on top of porous silicon sensors: a win-win situation

*Ruth F. Balderas-Valadez and Claudia Pacholski**

University of Potsdam, Institute of Chemistry, Karl-Liebknecht-Str. 24-25, 14476 Potsdam OT Golm, Germany

Corresponding author: cpachols@uni-potsdam.de

Experimental Details

Chemicals and Materials

Silicon wafer doped with Boron (p-type, resistivity <0.001 Ω) were obtained from Sil'Tronix Silicon Technology (France). Hydrofluoric acid (48%), 2-propanol, methanol and toluene were purchased from Merck. APTES, sulfuric acid, sodium hydroxide, *N*,*N'*-methylene bisacrylamide (BIS), sodium dodecyl sulfate, potassium peroxodisulfate, glass coverslips (24 x 24 mm) and ethanol 99.9% were supplied by Carl Roth GmbH + Co. KG (Germany). $HAuCl4 \cdot 3\ H_2O$ (99.99%) was obtained from Alfa Aesar (Thermo Fisher (Kandel) GmbH, Germany). *N*-isopropylacrylamide (NIPAM), hydroxylamine hydrochloride, protein A, PBS buffer, bovine serum albumin, and acetic acid were supplied by Sigma. Styrene was obtained from VWR.

Preparation of plasmonic nanohole arrays

Plasmonic nanohole arrays were fabricated using a modified method from Quint and Pacholski which is based on a combination of colloidal lithography and gold deposition.[1] In this work, polystyrene@poly-*N*-isopropylacrylamide (PS@polyNIPAM) core-shell particles were utilized instead of pure poly-*N*-isopropylacrylamide microgels for preparing the colloidal mask. The PS@polyNIPAM core-shell particles were synthesized according to Kim *et al.*[2] and purified by centrifugation, decantation and redispersion in MilliQ water for at least 3 times.

To deposit a loosely packed hexagonally ordered colloidal array on top of glass cover slips, the purified PS@polyNIPAM core-shell particle dispersion was mixed with ethanol (99.8 %) in a volumetric ratio of 1:1. 10 µL of the resulting mixture was spread over a hydrophilic glass coverslip (24 x 24 mm, cleaned with piranha solution (3:1 (v:v) mixture of concentrated H_2SO_4 : H_2O_2 (30%)) for at least 1 h) which was afterwards slowly dipped into a MilliQ water bath (500 mL water in a crystallizing dish with an internal diameter of 11 cm) at RT. Thereby, PS@polyNIPAM particles were transferred to the air/water interface. After addition of 4 µl of sodium dodecyl sulfate (10 weight% in MilliQ water) a well-ordered hexagonal array of PS@polyNIPAM particles is formed which can be lifted-off the interface using highly hydrophilic glass coverslips (pre-cleaned in piranha solution: conc. H_2SO_4 : H_2O_2 (30%) 3:1 (v:v)). Best results were achieved by slowly immersing the glass cover slip at an angle of ~60 ° to the water surface and slowly removing it from the water at the same angle. Afterwards, the samples were dried in air at RT. The extraordinary properties of the

PS@polyNIPAM particles led to a loosely packed, but well-ordered hexagonal colloidal array which was directly utilized as mask for the deposition of a gold film.

For providing adhesion between glass surface and gold film, the glass coverslips decorated with PS@polyNIPAM core-shell particles were first functionalized with APTES. Briefly, 30µL of APTES were mixed with 2 ml of isopropanol. The samples together with a small container filled with 150 µL of diluted APTES were placed in a 150 mL glass jar. The jar was closed and placed inside a preheated oven at 110°C for 1 h. The samples were taken out and washed thoroughly with MilliQ water. After drying under a stream of N_2 the samples were baked once more at 110°C for 1 h. Then, a thin layer of gold (~5 nm thickness) was deposited on the samples using a Quorum technologies Ltd. (Q150R ES) sputter coater.

To remove the colloidal mask the samples were first submerged in toluene at 8°C for at least 2 h and subsequently ultrasonicated in toluene at ~ 13°C for 60 min. After drying the samples in air the same procedure was carried out using a methanol : MilliQ water mixture (95:1, v:v) as immersion medium instead of toluene. The samples were washed with ethanol and dried in a stream of N_2 leaving behind a thin gold layer patterned with holes in a hexagonal array. The thickness of the gold layer was increased to the desired height using electroless gold deposition. In an open square plastic box (2.6 x 2.6 x 0.8 cm) 2 ml of an aqueous solution of $HAuCl_4$ * $3H_2O$ (5 mg/mL) and 33 µl of an aqueous solution of hydroxylamine hydrochloride (5.54 mg/ml) were mixed. The samples were placed face down onto the solution. The reaction was carried out for 30 min on an orbital shaker (slow motion). The resulting plasmonic nanohole arrays were removed from the gold salt solution, rinsed with MilliQ water and dried in air.

Assembly of hybrid sensor composed of porous silicon and plasmonic nanohole arrays

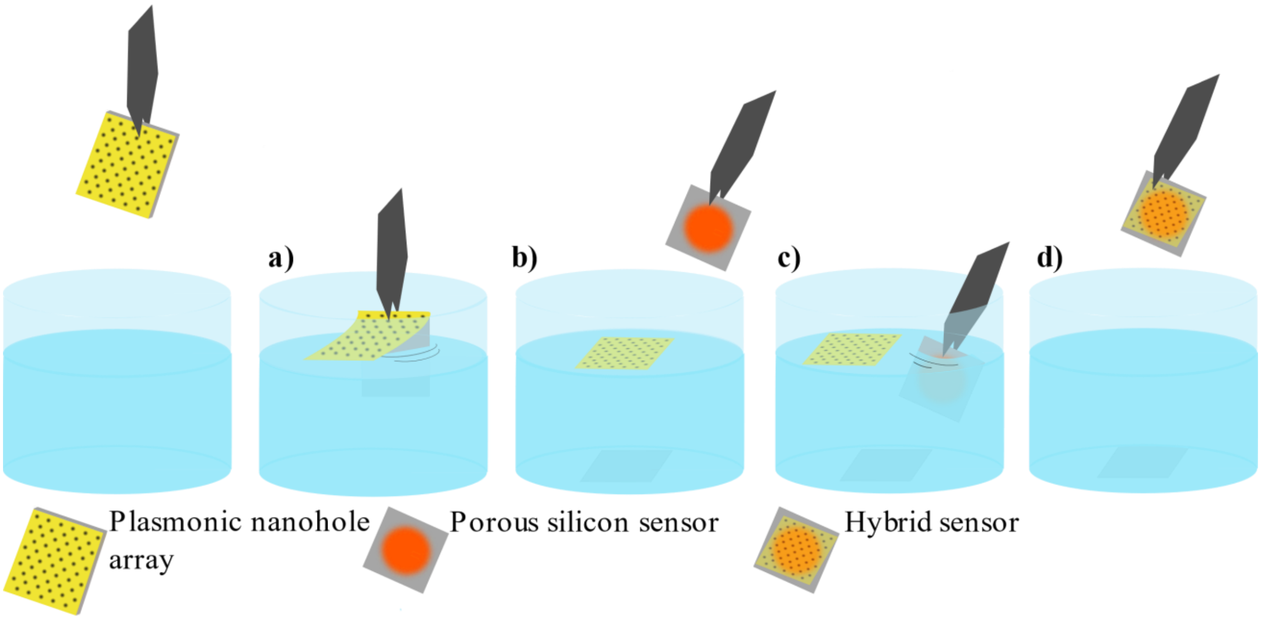

Figure S1. Schematic representation of the fabrication of the hybrid sensor. The plasmonic nanohole array was placed in 0.1 M aqueous NaOH solution overnight to break the covalent bonds between glass substrate and gold film provided by APTES. a) The nanohole array on the glass substrate is immersed vertically in the MilliQ water bath. b) The nanohole array tends to float on the water surface due to its light weight, while the glass substrate sinks to the bottom of the water bath. c) The porous silicon substrate is immersed in water and placed directly under the gold layer. d) Finally, the porous silicon substrate is lifted out of the water together with the nanohole array. The sample is dried vertically in air and tempered at 45°C for 24 hours.

Similar approaches have already been described several times in the literature.[3–5]

Supporting figures:

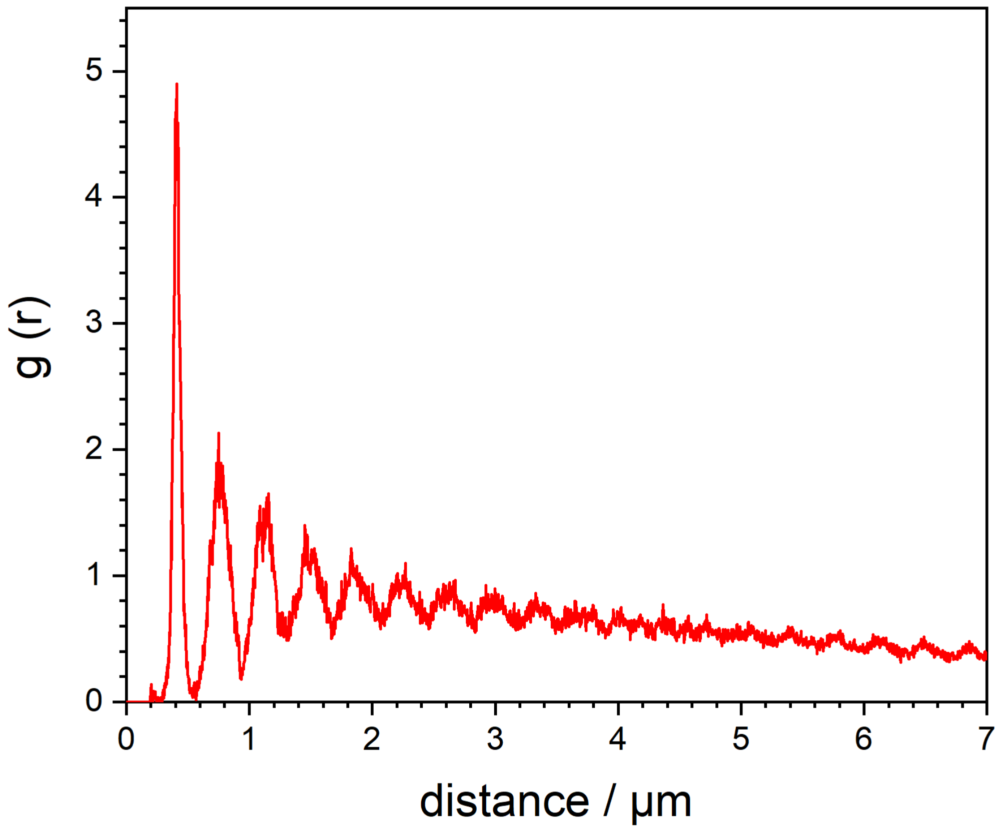

Figure S2. Radial distribution function of a plasmonic nanohole array. The center-to-center distances of the holes are well-defined and an averaged value can be obtained from the first sharp maximum (413 nm ± 2 nm). The diameter of hexagonally ordered domains can be estimated at 10 lattice constants.

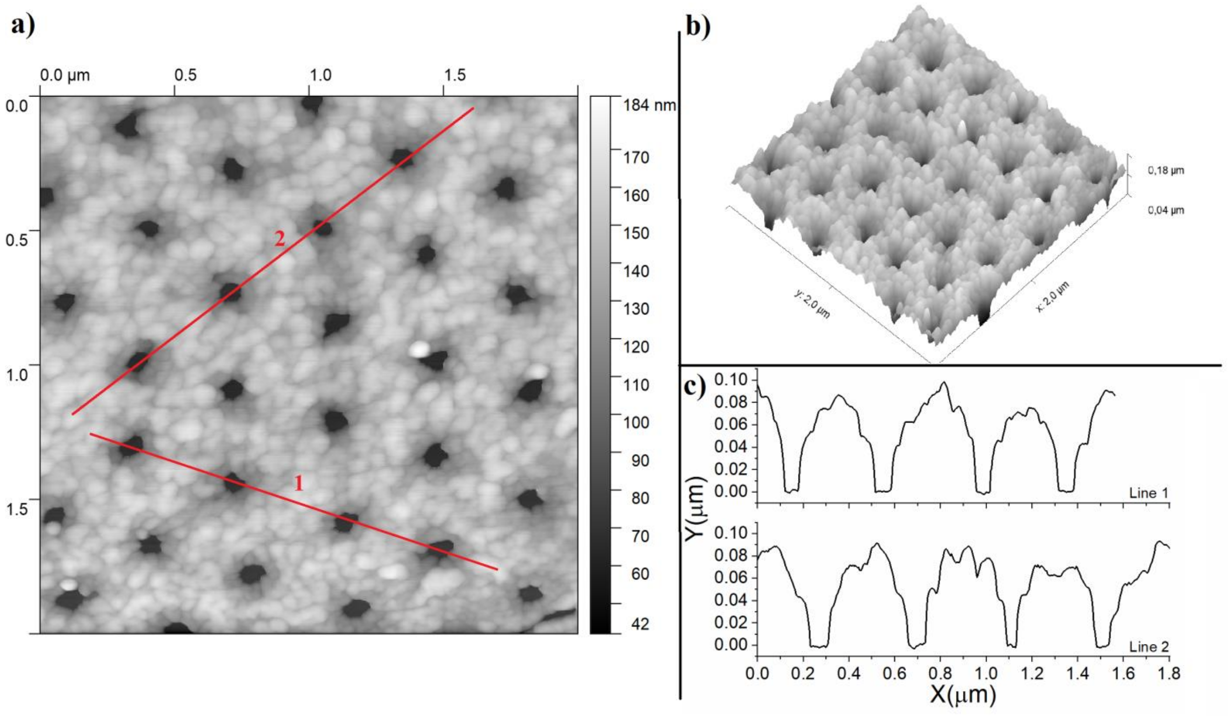

Figure S3. Characterization of plasmonic nanohole arrays using atomic force microscopy (AFM): a) topographical AFM image (size: 2 µm x 2 µm), b) three-dimensional representation of the topographical AFM image c) determination of gold film thickness: cross-sections taken at the indicated red lines in a).

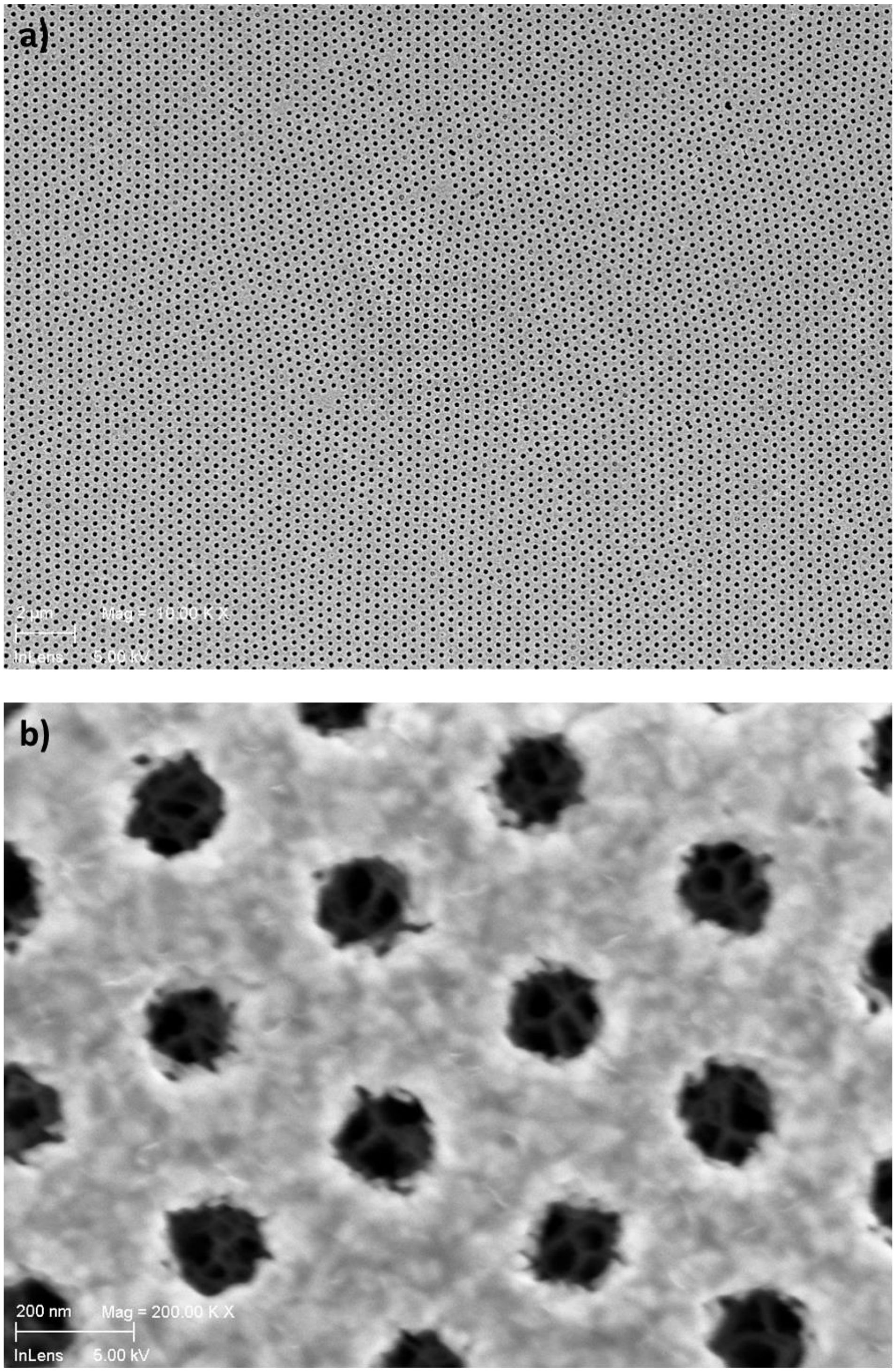

Figure S4. SEM images of a periodic hole array in a metallic film on top of porous silicon monolayers: a) overview SEM image demonstrating the homogeneity of the plasmonic nanohole array after transfer onto porous silicon and b) SEM image taken at high magnification showing the porous silicon pores underneath the plasmonic nanohole array.

Transmittance/ reflectance comparison

The plasmonic nanohole arrays are fabricated on a glass substrate. To obtain a reference sample consisting of a plasmonic nanohole array on a Si substrate, we used the same procedure that was used to transfer the plasmonic nanohole array onto porous silicon substrates. More specifically, we used a piece of polished silicon wafer (the same type used to prepare the porous silicon samples) oxidized at 600°C for 1 hour and functionalized with APTES (as described in the section on PSi preparation). Figure S5 a) shows the reflectance spectrum of the plasmonic nanohole array recorded in air after transfer to the silicon wafer. Obviously, the surface plasmon resonance (SPR) of the plasmonic nanohole array cannot be detected with the optical setup used before. It is now important to note that the SPR property has not disappeared, but has shifted to the UV region. This is probed by changing the refractive index of the surrounding medium from air to an aqueous solution of 50% of sucrose (w/w) as is shown in figure S5 b). Due to the increase of the refractive index of the surrounding medium the LSPR shifts to the visible range (~593 nm) as is highlighted by the red arrow. Therefore, transmittance spectra of the plasmonic nanohole arrays on glass substrates were recorded in order to determine the sensitivity of this structure.

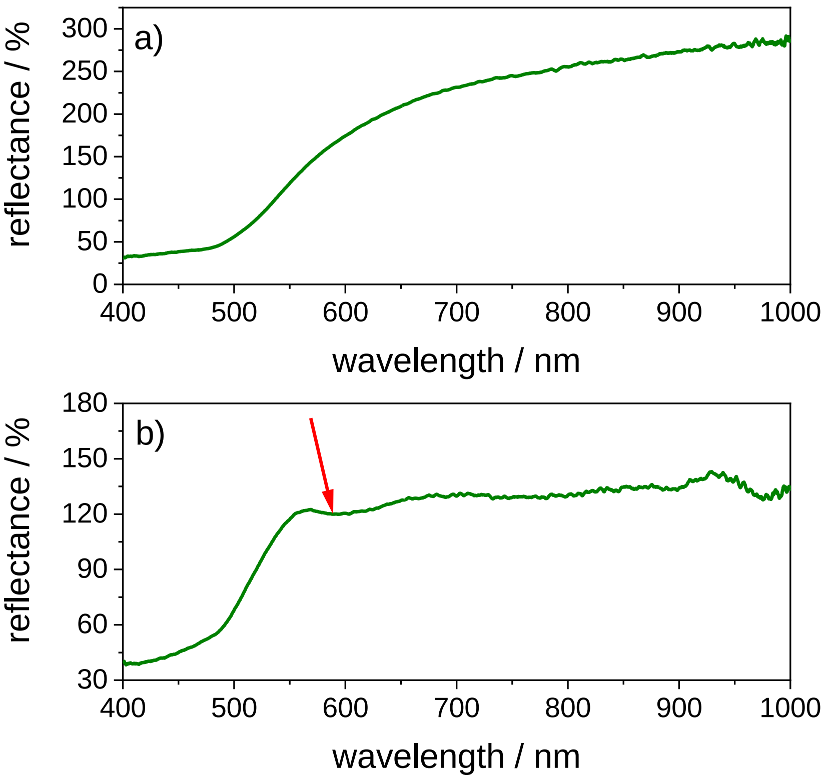

Figure S5. Reflectance spectra of a plasmonic nanohole array deposited on a polished silicon wafer: a) Spectrum recorded in air. b) Spectrum recorded after immersing the structure in 50 wt% sucrose in MilliQ water.

Determination of sensitivities – raw data

Figure S6 shows the raw data of the EOT shifts of 3 porous silicon sensors and 3 hybrid sensors. These shifts were calculated as the difference of the EOT of the sensors immersed in different aqueous sucrose solutions (EOT_{sn}) minus the EOT of the sensors immersed in water (EOT_{w}).

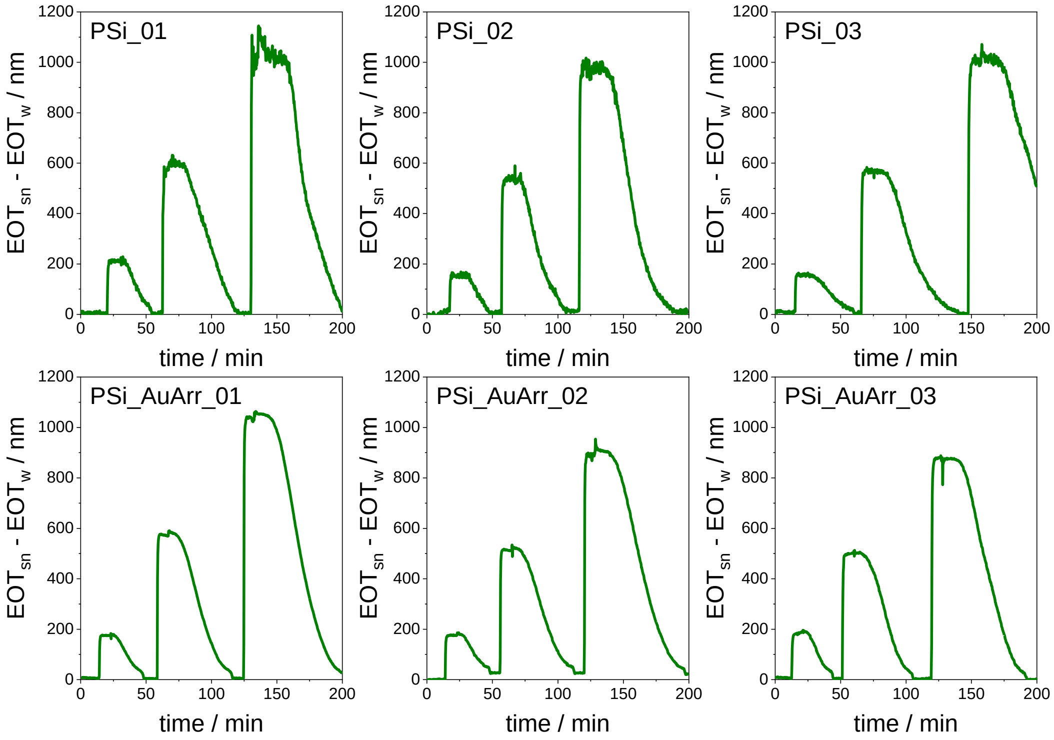

Figure S6. EOT shifts of 3 porous silicon sensors (Psi_01-Psi_03) and 3 hybrid sensors (PSI_AuArr_01- PSI_AuArr_03).

The intensity of the EOT peak was also used as a transduction signal. The raw signals in sensors with and without plasmonic nanohole array on porous silicon monolayers are shown in Figure S7. The change in intensities was calculated as the intensity of the EOT peak of the sensors immersed in sucrose solutions ($IntEOT_{sn}$) minus the intensity of the EOT peak of the sensors immersed in water ($IntEOT_{w}$).

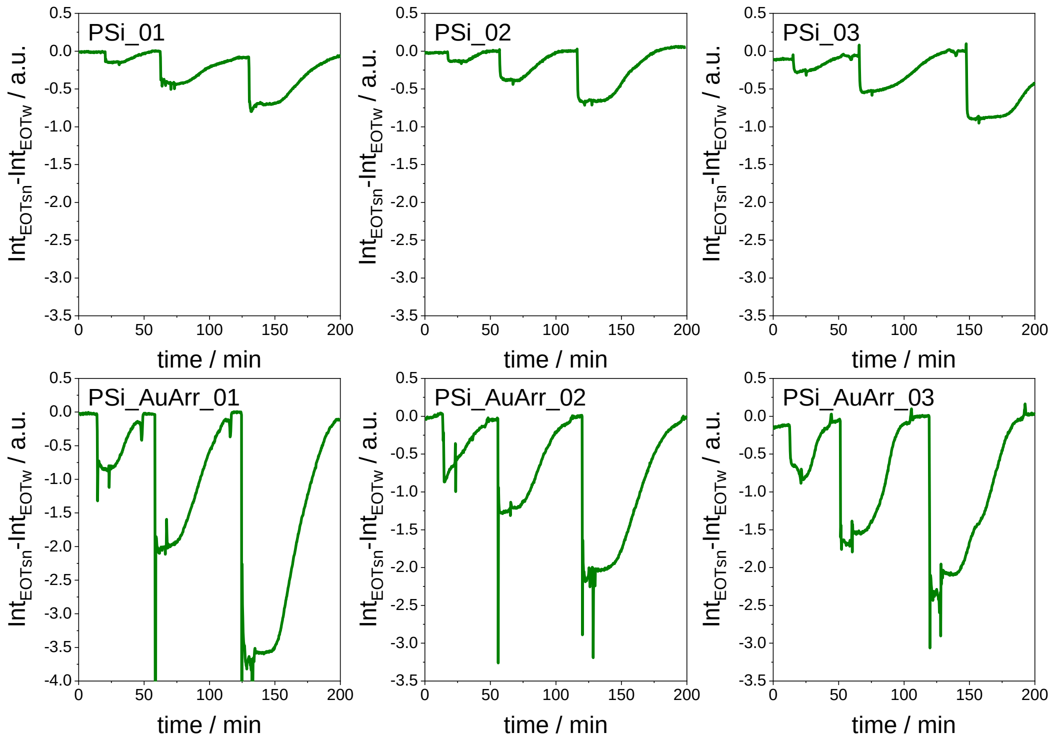

Figure S7. EOT intensity changes of 3 porous silicon sensors (Psi_01-Psi_03) and 3 hybrid sensors (PSI_AuArr_01- PSI_AuArr_03).

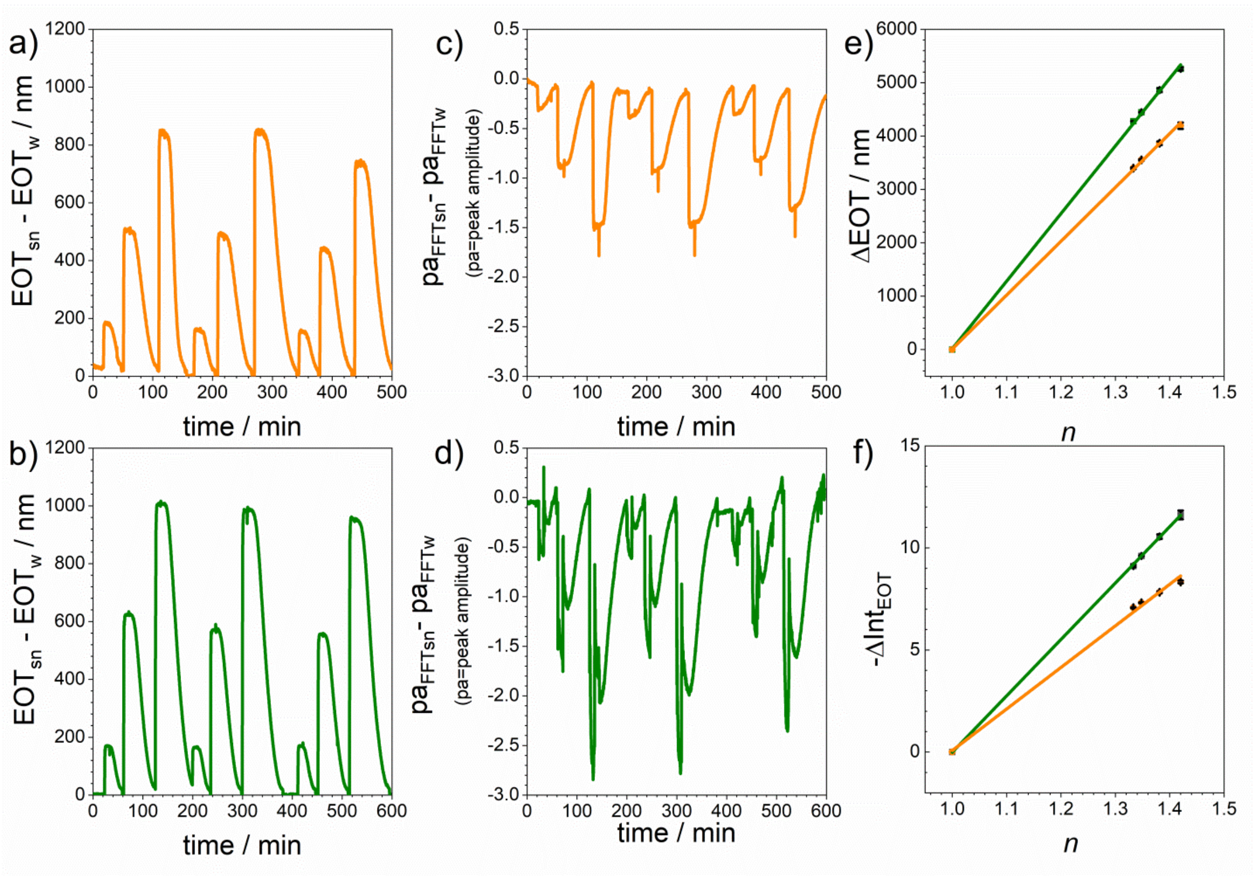

Figure S8. Reproducibility of the optical response of one sensor (porous silicon (orange) and hybrid sensor (green)) for different sucrose solutions. Error bars represent the standard deviation for changes in the optical signal corresponding to one sucrose solution (shown in black, almost invisible due to their small values).

The SPR position shifts of hybrid sensors and plasmonic nanohole arrays on glass are compared and shown in Figure S9. The spectral shifts were calculated as the wavelength of the SPR of the sensors immersed in aqueous sucrose solution (λsn) minus the wavelength of the sensor exposed in water (λw).

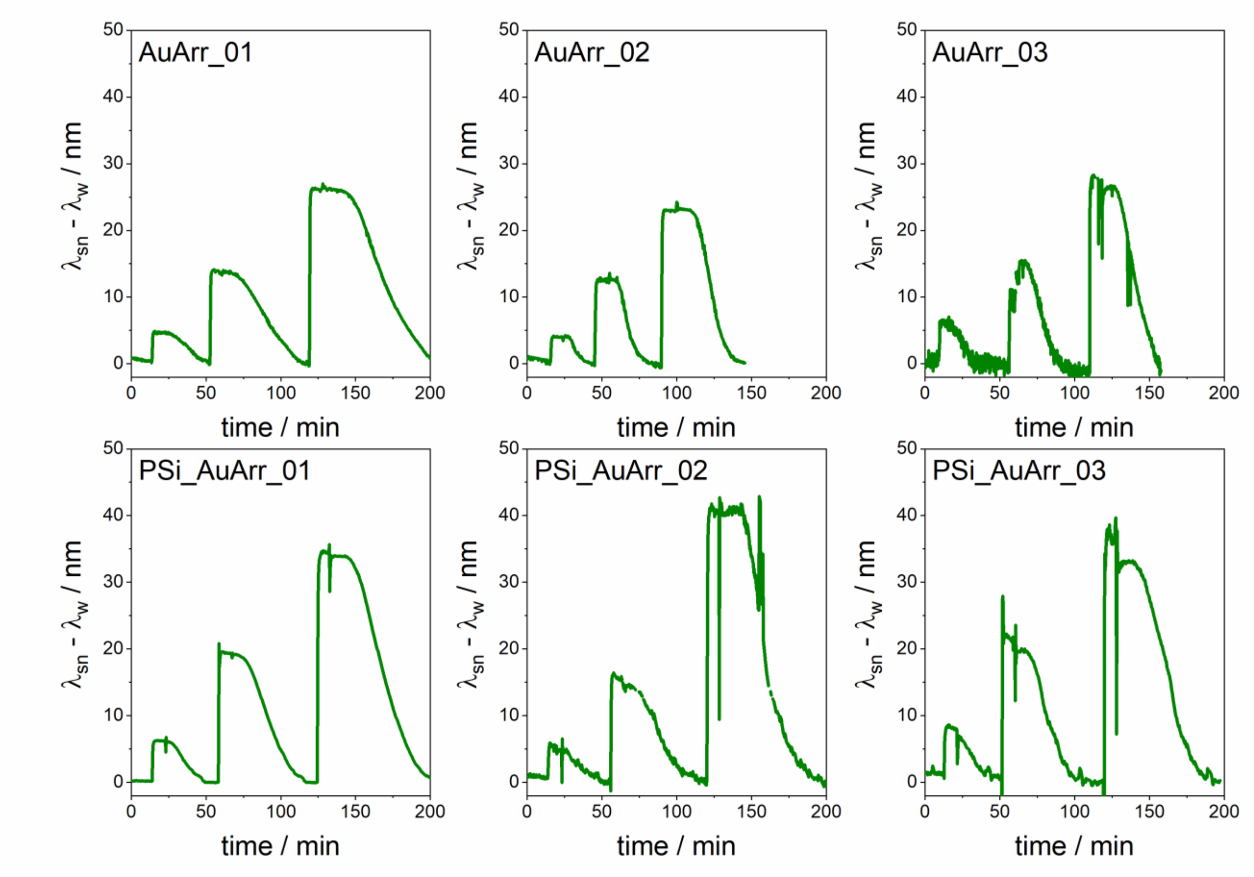

Figure S9. Changes in the spectral position of the SPR of plasmonic nanohole arrays on glass substrates (AuArr_01- AuArr_03) and plasmonic nanohole arrays on porous silicon monolayers (PSI_AuArr_01- PSI_AuArr_03).

Dual mode sensing experiment

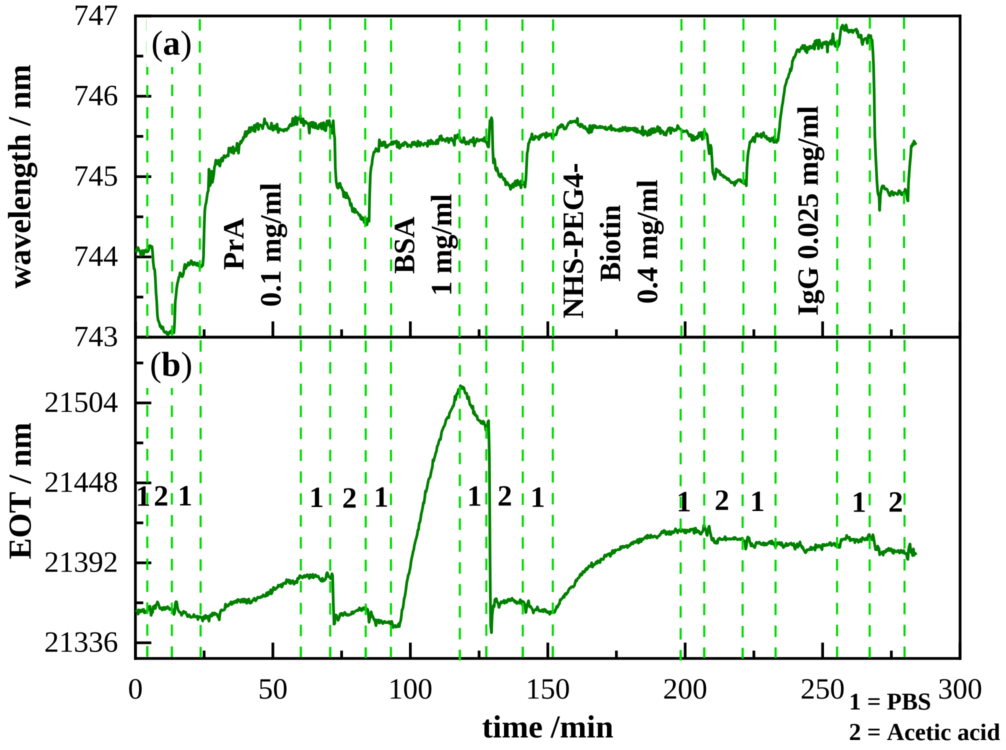

Figure S10. Dual-mode sensing with a hybrid sensor consisting of a plasmonic nanohole array on porous silicon monolayers: Repetition with a different hybrid sensor.

References

(1) Quint, S. B.; Pacholski, C. A Chemical Route to Sub-Wavelength Hole Arrays in Metallic Films. *J. Mater. Chem.* **2009**, *19* (33), 5906–5908. https://doi.org/10.1039/B910892K.

(2) Kim, J.-H.; Ballauff, M. The Volume Transition in Thermosensitive Core-Shell Latex Particles Containing Charged Groups. *Colloid Polym. Sci.* **1999**, *277* (12), 1210–1214. https://doi.org/10.1007/s003960050512.

(3) Lipomi, D. J.; Martinez, R. V.; Kats, M. A.; Kang, S. H.; Kim, P.; Aizenberg, J.; Capasso, F.; Whitesides, G. M. Patterning the Tips of Optical Fibers with Metallic Nanostructures Using Nanoskiving. *Nano Lett.* **2011**, *11* (2), 632–636. https://doi.org/10.1021/nl103730g.

(4) Du, B.; Ruan, Y.; Yang, D.; Jia, P.; Gao, S.; Wang, Y.; Wang, P.; Ebendorff-Heidepriem, H. Freestanding Metal Nanohole Array for High-Performance Applications. *Photonics Res.* **2020**, *8* (11), 1749. https://doi.org/10.1364/PRJ.397409.

(5) Wu, H.; Li, C.; Zhao, Z.; Li, H.; Jin, Y. Free-Standing Monolayered Metallic Nanoparticle Networks as Building Blocks for Plasmonic Nanoelectronic Junctions. *ACS Appl. Mater. Interfaces* **2016**, *8* (3), 1594–1599. https://doi.org/10.1021/acsami.5b11805.
